# Kinking of Frozen Elephant Trunk Hybrid Prostheses: Incidence, Mechanism, and Management

**DOI:** 10.3389/fcvm.2022.912071

**Published:** 2022-04-27

**Authors:** Fatima Kayali, Sara Qutaishat, Matti Jubouri, Rohan Chikhal, Sven Z. C. P. Tan, Mohamad Bashir

**Affiliations:** ^1^School of Medicine, University of Central Lancashire, Preston, United Kingdom; ^2^School of Medicine, University of Jordan, Amman, Jordan; ^3^Hull York Medical School, University of York, York, United Kingdom; ^4^Barts and The London School of Medicine and Dentistry, Queen Mary University of London, London, United Kingdom; ^5^Vascular & Endovascular Surgery, Velindre University NHS Trust, Health Education & Improvement Wales (HEIW), Cardiff, United Kingdom

**Keywords:** Frozen Elephant Trunk, hybrid prosthesis, kinking, aortic dissection, aortic aneurysm

## Abstract

**Introduction:**

Kinking of the Frozen Elephant Trunk (FET) stent graft is one of the most devastating complications of the FET procedure. It can present post-operatively with reduced arterial pressures in the lower limbs and intermittent claudication. However, it can also be visualized intra-operatively by the surgeons. Unresolved kinking of the stent graft can result in intraluminal thrombus formation and subsequent multi-organ septic emboli.

**Aims:**

The main scope of this review is to collate, summarize and present all the evidence in the literature on kinking of FET stent grafts.

**Methods:**

We carried out a comprehensive literature search on multiple electronic databases including PubMed, EMBASE, Ovid, and Scopus to collate all research evidence on the incidence, mechanism, and management of FET graft kinking.

**Results:**

Incidence of kinking is variable, ranging from 0% to 8% in the literature, with varying rates associated with each stent graft type. The Thoraflex Hybrid^TM^ prosthesis seemed to be the most commonly used and superior graft, and out of all the 15 cases of kinking reported in the literature, 5 (33.3%) were associated with just the Frozenix graft which had the highest incidence. There are multiple theories regarding the mechanism of kinking, including the direction of blood flow, the length of the stent grafts used, and the position of the prosthesis in relation to the flexure of the aorta. Multiple reparative management techniques have been suggested in the literature and include total endovascular repair, open repair, balloon dilatation, and deploying a second stent graft.

**Conclusion:**

Graft kinking is one of the most critical complications of the FET technique. Its life-threatening sequelae warrant appropriate follow-up of these patients post-operatively, in addition to time management if kinking is suspected. Given the limited evidence in the literature, future studies should incorporate graft kinking into their outcomes reporting.

## Introduction

Pathologies involving the aortic arch and descending thoracic aorta, such as thoracic aortic aneurysms and dissections, pose a prominent challenge to cardiovascular surgeons, carrying a significant risk of morbidity and mortality. In 1983, Borst was the first to describe the Elephant Trunk technique (ET), a two-stage procedure to repair complex thoracic aorta pathologies that were limited by the fact that many patients did not make it to the second operation (1–3). Later on, in 2003, these two surgeries were combined into a single-step hybrid procedure that was named the ‘Frozen Elephant Trunk' (FET). In FET, the proximal aspect of the aortic arch is reconstructed surgically and the distal is repaired endovascularly during the same hybrid operation (1). Multiple FET hybrid prostheses are currently available commercially and globally, including the Frozenix J Open Graft, Cronus, E-Vita, and Thoraflex Hybrid. Each type has its unique design, thus they each vary in terms of technical aspects, as well as their clinical outcomes, with Thoraflex Hybrid showing superior results, both regionally and globally (4).

Despite excellent results yielded by the FET technique, multiple complications associated with its use have been reported. One of the major drawbacks to FET is its array of potential neurological complications, such as spinal cord injury (SCI), paraplegia, and stroke (5, 6). Other complications reported include coagulopathy, postoperative bleeding, recurrent nerve palsy, vocal cord paralysis, renal failure necessitating dialysis, prolonged mechanical ventilation, and multi-organ failure (5, 7–11). In-hospital and 30-day mortality rates associated with FET range from 0 to 17.2% and 0 to 18.2%, respectively (11). In a retrospective study by Kreibich et al. (12), 35 patients (33% of the sample) had FET-related complications necessitating reintervention, out of which 2 patients (6%) had significant kinking of the stent graft.

The presentation of FET stent graft kinking is variable, and while it may be simply detected on follow-up imaging after FET with CT angiography (CTA), in many cases it may be distinguished by low arterial pressure in the lower limbs relative to upper limbs (13). Other case reports have stated that kinking may also manifest as intermittent claudication (14). Additionally, studies have mentioned that the diagnosis of endostent graft kinking can occur both intra- and post-operatively (13–15).

Due to the underlying mechanism of this pathology, which will be discussed in detail in this review, kinking is associated with an array of life-threatening consequences for the FET patient. Turbulence and stasis of blood flow are thought to encourage intraluminal thrombus formation secondary to the stenosis; this can have devastating outcomes, such as septic embolism and multi-organ ischemic damage (16). Subsequently, appropriate preventative measures and thorough follow-ups are essential.

This review aims to collate, summarize and present the evidence in the literature on the incidence, mechanism, and management of FET stent graft kinking.

## Incidence

Incidence of kinking following the FET procedure is seldom reported in the literature. As seen in Table 1, in the limited studies found, the variable incidence of kinking was reported, ranging from 0% in Thoraflex Hybrid and E-Vita and up to 8% in a branched one-piece graft by Yuhengjia Sci-Tech Co. Ltd, Beijing, China (17, 18).

**Table 1:** Overall summary of the studies reporting on FET graft kinking, the type of graft used, and the incidence of kinking.

**References**	**Year**	**Type of FET Graft**	**Incidence of kinking n (%)**
Kreibich et al. (12)	2020	Thoraflex Hybrid	2/35 (5%)
Uchida et al. (13)	2019	Frozenix	2 cases
Morisaki et al. (14)	2018	Frozenix	2 cases
Nakagawa et al. (15)	2022	120-mm-long woven Dacron graft with a Gianturco Cook-Z stent (Cook Medical, Inc., Bloomington, Ind) attached only to the distal end	1 case
Imamura et al. (16)	2021	Hand-made FET (UBE graft, UBE Industries, Ltd., and Z-shaped stent, William Cook)	1 case
Detter et al. (17)	2019	E-Vita Open Plus (Jotec, Hechingen, Germany) and Thoraflex Hybrid	0/92 (0%)
Shen et al. (18)	2012	A branched 1-piece graft (Yuhengjia Sci-Tech Co. Ltd, Beijing, China).	3/38 (8%)
Shrestha et al. (19)	2014	Vascutek Siena	0/179 (0%)
Toda et al. (20)	2009	4-branched arch graft (Hemashield Platinum, MAQUET Cardiovascular LLC, Wayne, NJ)	0/111 (0%)
Spielvogel et al. (21)	2005	Handmade and graft by Boston Scientific, Natick, MA	0/109 (0%)
Uchida et al. (22)	2016	Frozenix	1/60 (2%)
Ho et al. (23)	2021	E-Vita Open NEO	0/3 (0%)
Ouzounian et al. (24)	2020	Homemade, Thoraflex Hybrid (Vascutek, Glasgow, Scotland), Evita Open Plus (Jotec, Hechingen, Germany/CryoLife, Kennesaw, GA, USA), and Cook Hybrid (Cook Medical, Bloomington, IN, USA) graft.	0/167 (0%)
Öz et al. (25)	2021	Thoraflex Hybrid	1 case
Baraki et al. (26)	2007	Hybrid prosthesis (Chavan-Haverich endograft, Curative GmbH, Dresden, Germany).	1/39 (3%)
Karck et al. (27)	2005	Hybrid prosthesis [“Chavan–Haverich” (CH) endograft, Curative GmbH, Dresden, Germany]	1/22 (5%)

Thoraflex Hybrid was one of the most commonly used devices with the best outcomes when it comes to stent graft kinking, as demonstrated in Table 1. The aforementioned study by Kreibich et al. (12) reported 2 cases of postoperative graft kinking in 107 patients (2%) who underwent total arch replacement (TAR) with FET using Thoraflex Hybrid, both of whom required reintervention. However, the association between stent graft kinking and aortic reintervention was not statistically significant (*p* = 1.00) (12). Additionally, Öz et al. (25) reported one case of kinking of this prosthesis post-FET. Here, the patient's 2- and 2.5-year follow-up CTA revealed graft kinking with proximal migration of the endograft portion of the FET, resulting in new thrombus formation, thus necessitating endovascular reintervention which was performed successfully. Lastly, 2 relatively large studies using Thoraflex Hybrid did not observe any cases of graft kinking amongst their study population of 92 and 167 patients, respectively (17, 24).

In a similar trend to Thoraflex Hybrid, no cases of kinking were reported in association with the E-Vita device. Ho et al. (23) assessed the use of the branched version of E-vita Open NEO in 3 patients that underwent TAR using FET for type B aortic dissection with an arch aneurysm. In this case series, a post-operative CT aortogram was done that showed no kinking in all 3 patients (23). Additionally, in a study by Detter et al. (17), none of the 92 patients who also underwent TAR with FET using the E-Vita Open Plus hybrid prosthesis (HP) developed kinking.

Interestingly, it is worth noting that, unlike Thoraflex Hybrid, studies on E-Vita seldom featured graft kinking in their design and outcomes reporting. On the other hand, more FET studies using Thoraflex Hybrid included kinking in their data collection methodology for larger patient populations, thereby increasing the reliability of the evidence supporting its superiority.

Incidence of graft kinking with the Frozenix HP was more variable, and overall, the highest, which represents a great concern over the safety and effectiveness of this FET graft. Uchida et al. (13) reported 2 cases of TAR with FET using the Frozenix J Open Graft, both of whom developed stent graft kinking which was related to the stent graft's structural property (13). Here, during the first case, specifically, after the Frozenix HP was inserted into the downstream aorta and its distal anastomosis completed, high cardiopulmonary bypass (CPB) flow-line pressure (300 mm Hg) and low femoral arterial pressure were noted, suggesting FET graft obstruction. Transoesophageal echocardiography (TOE) showed kinking of the Frozenix HP. Similarly, during the second case, Frozenix graft kinking was luckily also discovered intraoperatively. Additionally, 2 cases of Frozenix kinking post-FET for acute Type A aortic dissection were reported by Morisaki et al. (14). In the first case, after emergency TAR with FET, the patient developed intermittent claudication post-operatively, which on CTA showed to be kinking between the non-stent and stent parts of the Frozenix HP that required endovascular reintervention. Patient 2 experienced decreased blood pressure in the lower body and intermittent claudication following a reimplantation root procedure and translocated TAR with the FET. On CTA, this was also discovered to be kinking between the non-stent and stent parts of the Frozenix HP that also required endovascular reintervention (14).

Furthermore, a 2016 multicentre study in Japan set out to evaluate the incidence of major adverse outcomes in patients using the Frozenix stent graft. Here, 2% of patients developed kinking of this prosthesis intraoperatively (22). As aforementioned and illustrated in Table 1, a total of 15 cases of kinking were reported out of which 5 (33%) were associated with Frozenix. The high incidence of Frozenix graft kinking must be taken into consideration very carefully in current and future practice.

Other FET devices which are less commonly used have also been described in the literature to be associated with kinking, a summary of these along with their kinking incidence can be found in Table 1 (15, 16, 18–21, 26, 27).

## Mechanism

Multiple factors have been suggested in the literature to cause kinking of the FET grafts. Firstly, the most significant consideration of surgical technique in total arch repair is in the direction of blood flow. In an evaluation of 30 years of experience involving 179 patients who underwent TAR with FET, Shrestha et al. (19) emphasized the significance of establishing antegrade blood flow through the FET graft. Retrograde flow is thought to cause kinking and unfolding of the distal graft at the descending thoracic aorta. This includes cannulation for cardiopulmonary bypass, where a retrograde direction of flow is encouraged (19).

Secondly, a longer prosthesis has been linked to an increased risk of developing kinking and a length of 7–8 cm is thought to be ideal (28). This was supported by Morisaki et al. (14). On the other hand, in a 10-year experience analysis, Toda et al. (20) reported no incidences of kinking in 111 patients, despite using a mean graft length of 15.9 ± 3.1 cm (10–22 cm), a significantly longer trunk than initially suggested. These findings are concomitant with a 5-year prospective study of 109 patients, where no kinking postoperatively was reported using the same longer graft length (21). The ideal length of the graft remains a topic of dispute, with longer grafts being unfavorable to some surgeons due to the increased risk of spinal cord ischemia (29). The fluttering of the non-stented distal end of the elephant trunk with the longer grafts also increases the risk of dislodging peripheral emboli, although this is more associated with conventional ET, and less with FET (3, 30, 31). However, it is important to note that the length of the prosthesis may be dictated by the patient presentation and characteristics as well as complications during the procedure, including aortic calcification, multifocal aneurysmal extensions, and concomitant aortic kinking (32). Graft measurements should ideally be done before surgery as during circulatory arrest the aorta collapses and shortens, impairing the accuracy of the measurements (33). Unlike the other aortic arch prostheses on the global commercial market, Thoraflex Hybrid represents a diverse HP option offering a variety of graft lengths and diameter combinations, allowing for a more personalized patient approach (4).

Thirdly, kinking can also occur if the stent graft is positioned at the flexure of the aorta (16). Uchida et al. (22), for instance, described one case of stent graft kinking at the border between the stented and non-stented part of the Frozenix HP. Unlike Frozenix, the Thoraflex Hybrid design does not feature a non-stent portion of the graft which does help prevent such cases of kinking between the distal anastomosis site and the stent (8, 14). Additionally, in a 2021 study involving the E-Vita HP, increased anastomotic space to the left subclavian artery was thought to reduce the risk of kinking. This wider gap was achieved by increasing the distance (20 mm) between the third-side branch of the prosthesis and the sewing collar (23). Moreover, manipulation of the graft architecture to prevent kinking was also discussed by Ma et al. (4). In some FET devices including Thoraflex Hybrid, it is possible to accustom the length of the side branches before forming an anastomosis or ordering a custom-made Thoraflex Hybrid prosthesis to accommodate specific patient anatomy with a 4-week delivery timeframe.

Concerning the graft-stent type and its association with kinking, only suggestions regarding the limitations of the prosthesis architecture were highlighted. For example, in comparison to other hybrid devices, Thoraflex Hybrid exerts a lower radial force onto the aortic walls. Although this is associated with positive outcomes in acute pathology, in chronic dissection this amplitude and force may cause compression and kinking of the stent, potentially introducing the need for ballooning and further intervention (24). Frozenix, on the other hand, was reported to cause functional obstruction of the graft due to impingement of the distal end of the prosthesis with the intimal flap. This is evident in the aforementioned case report by Morisaki et al. (14) which described 2 cases of FET obstruction secondary to kinking of Frozenix, these were thought to have occurred due to the sharp angulation of the aorta in both patients.

Interestingly, oversizing the graft has also been described as a prophylactic measure against kinking. For example, Chu et al. (34) recommend oversizing by 10–20%. It is thought that the curvature of the prosthesis, in combination with the low radial force within the aorta may cause proximal migration and subsequent kinking of the stent. Hence, in an attempt to counteract the vector forces causing the kinking, longitudinal support, in the form of interrupted interconnecting wires, has also been suggested in the literature (25, 35).

Finally, in addition to intraoperative technique changes and stent graft adjustments, the use of intravascular ultrasound during surgery has been recommended. This would ensure the correct placement of the wire that guides the prosthesis into the true lumen and would reduce the risk of kinking or depression of the stent (36). Further benefit yielded by the intraoperative ultrasound scan is highlighted in a different study by Baraki et al. (26), where failure to advance the guidewire and the introducer system resulted in perforation of the aorta. The Canadian Thoracic Aortic Collaborative also suggested using transoesophageal echocardiography to ensure correct stent placement with no kinking (24).

Multiple explanations of the mechanism of kinking following FET exist in the literature, often only based on an individual surgeon or single-center experiences, and further analysis of this topic on a wider scale is essential to implement further guidance.

## Management

Different approaches to the management of FET stent graft kinking have been described throughout the limited literature. One common way is to relieve kinking is by intervening endovascularly with thoracic endovascular aortic repair (TEVAR). Öz et al. (25) described “unfreezing” the kinked Thoraflex stent with TEVAR. Here, the authors inserted an extra-stiff guidewire into the securely cannulated kinked region, and with the use of an appropriately sized compliant balloon, balloon dilation was done twice (25). When inserting a guidewire into a kinked segment, caution should be taken as to not perforate the aortic wall and thus necessitating an extra open surgical repair (27).

By straightening out the kinked graft and realigning the FET, the surgeons were able to lengthen the graft, in addition to the proximal landing zone to achieve an Ishimaru landing zone 3 (25). In addition, Öz et al. (25) described placing the TEVAR where less curvature occurs, as in FET as well, to avoid malalignment and bird-beaking. As described earlier, because Thoraflex Hybrid enforces lower radial forces, during TEVAR, the FET was relined with Relay TEVAR endovascular graft to support its vertical arch. The Thoraflex Hybrid and Relay TEVAR, manufactured by Terumo Aortic, ensured that stent in-stent assessment was performed to provide reliable yet optimal clinical efficacy.

In addition to balloon dilatation, inserting a second stent graft can correct and reorient the FET, especially when significant stenosis is present (37). For instance, Nakagawa et al. (15) described the dilemma of correcting a structurally poor surgeon-modified prosthesis. Due to its weak architecture, with no graft-lining stent in the core of the graft, their surgeon-modified FET prosthesis became significantly kinked. Similar to Öz et al. (25), an extra-stiff guidewire was used to straighten the FET graft, however, balloon pre-dilatation was not necessary. A fenestrated stent graft was then delivered and placed at proximal aortic zone 0 (15).

Other reported cases of kinking identified intraoperatively were treated by pulling out of the non-stent part of the graft proximally and then re-anastomosing the distal part into a distal site. Uchida et al. (13) described performing this technique on a patient in Japan where the Frozenix stent graft was initially used. Similarly, for another patient, the same authors described taking out the Frozenix graft, trimming it, and re-anastomosing the graft more distally (13).

In addition to the previously mentioned management strategies, Kreibich et al. (12) investigated the use of open repair of the stent graft. This was often performed if there was evidence for extensive kinking or if other complications and comorbidities were present, such as connective tissue disorders, atherosclerotic plaques, or in the case of an inadequate distal landing zone (12).

It is important to take into consideration that, often, post-FET complications do not present in isolation, and the corrective management may vary from patient to patient. This can depend on the extent of the dissection or aneurysm, any concomitant aortic pathology, and the presence of other complications (12, 13).

In addition to corrective procedures or surgery to repair the kinked stent, adjunctive treatment to manage the complications of the kinking may also be necessary. For instance, this can occur where post-FET kinking is seen with evidence of thrombosis on CTA. As the kinking gets progressively worse, so can the stent occlusion. As a result, treatment with direct oral anticoagulation can be initiated before further surgical intervention, to decrease the likelihood of thromboembolic events perioperatively (25).

Despite the limited literature, sufficient evidence is available to support the continued and increasing application of Thoraflex Hybrid as well as the necessary re-evaluation of Frozenix's safety and effectiveness as a FET HP. As mentioned previously, although the single-step FET procedure does yield excellent results, particularly positive aortic remodeling, there remain several complications associated with it, only one of which is graft kinking. These were addressed in 2 recent studies by Tan et al. (38) and Jubouri et al. (39) who proved the efficacy of FET and the well-established Thoraflex Hybrid as the superior FET HP available commercially.

## Conclusion

FET graft kinking is a serious complication of this hybrid procedure that must be approached swiftly but attentively, as although it is rare in incidence, it can lead to devastating results. Surgeons must carefully consider their choice of FET graft as well as their surgical technique while bearing in mind their management strategy in case of kinking occurrence intra- or post-operatively. Finally, future FET studies must incorporate graft kinking into their outcomes reporting.

## Author Contributions

FK, SQ, MJ, and RC were involved in literature review design, literature search, and manuscript writing. ST and MB were involved in manuscript revision. All authors contributed to the article and approved the submitted version.

## Conflict of Interest

The authors declare that the research was conducted in the absence of any commercial or financial relationships that could be construed as a potential conflict of interest.

## Publisher's Note

All claims expressed in this article are solely those of the authors and do not necessarily represent those of their affiliated organizations, or those of the publisher, the editors and the reviewers. Any product that may be evaluated in this article, or claim that may be made by its manufacturer, is not guaranteed or endorsed by the publisher.
